# ERAS vs. Traditional Protocol in Patients Who Had Radical Cystectomy with Ileal Conduit: A Retrospective Comparative Analysis of 182 Cases

**DOI:** 10.1155/2022/7335960

**Published:** 2022-02-28

**Authors:** Ahmet Semih Guleser, Yasar Basaga

**Affiliations:** ^1^Urology Department, Yeni Yuzyil University, İstanbul, Turkey; ^2^Urology Department, Nisantasi University, İstanbul, Turkey

## Abstract

**Objective:**

To examine the effects of ERAS protocol application on hospital stay, postoperative antibiotic use, and gastrointestinal recovery time in radical cystectomy patients with ileal conduit.

**Materials and Methods:**

This retrospective study included 182 patients (112 traditional vs. 72 ERAS) who underwent radical cystectomy (RC) with ileal conduit between November 2017 and December 2020. Patients were compared in terms of time to start enteral feeding (SEF), length of hospital stay (LOS), time to first stool, duration of postoperative intravenous antibiotic use, postoperative ileus rate, and serum albumin levels.

**Results:**

The traditional and ERAS groups contained 112 and 72 patients, respectively. LOS (14.79 ± 6.44 vs. 10.44 ± 4.64 days, *p*=0.003), first stool time (4.43 ± 2.39 vs. 2.89 ± 1.81 days, *p*=0.011), and duration of postoperative intravenous antibiotic use (8.79 ± 5.17 vs. 4.61 ± 4.90, *p*=0.004) were to be found significantly shorter in the ERAS group.

**Conclusion:**

According to the results of this study, the ERAS protocol shortened the length of hospital stay, duration of antibiotic use, and time of first stool in patients who underwent RC with ileal conduit.

## 1. Introduction

Bladder cancers are the second most common of the genitourinary system. Approximately 20–40% of them are muscle invasive at the time of diagnosis [1]. Radical cystectomy (RC) and pelvic lymph node dissection are standard treatment modalities for muscle-invasive bladder tumors [2]. The primary goal of bladder cancer treatment is to minimize mortality and morbidity while obtaining the best oncological results [3]. Fortunately, morbidity and mortality from RC have decreased in recent years due to advances in surgical technique, anesthesia, and postoperative procedures [4].

Although various methods are used for urinary diversion, the ileal conduit is currently the most preferred method [5]. Early complications such as nausea, vomiting, fever, and ileus have been reported in patients after the ileal conduit procedure, however [6]. These complications affect the length of hospital stay and increase the cost of the operation.

In the past, the lack of a standard protocol in postoperative healthcare resulted in different approaches to patients, such as a postoperative bowel rest period and/or a preoperative bowel cleaning, and pain management decisions differed for each surgeon [7]. Today, enhanced recovery after surgery (ERAS) is in place, which is the concept of multimodal, perioperative interventions to improve postoperative outcomes. ERAS protocol consists of 21 topics that concern the preoperative, intraoperative, and postoperative periods. The protocol is based on anesthesia, analgesia, perioperative fluid management, nutrition, early mobilization, and shortening the healing process by reducing the metabolic trauma caused by surgery [8]. Recently, studies in the field of urological surgery have started with the ERAS protocol. It has been shown that the length of hospital stay (LOS) and the rate of development of ileus are statistically significantly less in patients who underwent the ERAS protocol compared to those using the traditional approach [9–11]. Although more studies are required, no significant difference was observed in terms of anastomotic leaks, peritonitis, or other complications [12].

Our study aimed to compare the start time of enteral feeding (SEF), the first stool time, LOS, duration of postoperative antibiotic use, rates of ileus, and serum albumin values of patients with ileal conduit under the ERAS protocol with those who underwent a traditional protocol.

## 2. Materials and Methods

### 2.1. Patient Selection and Data Collection

Approval was obtained from the ethics committee with the date 28/04/2021 and number 380. The retrospective study included patients who underwent RC with ileal conduit between November 2017 and December 2020. Patients who were selected for diversion methods other than the ileal conduit, such as ureterocutaneostomy or orthotopic bladder, were excluded from the study. Demographic characteristics, including age, body mass index, gender, and clinical stage, and clinical characteristics, including plasma albumin level (g/L), SEF (day), LOS (day), duration of postoperative antibiotic use, and first stool time, were recorded for each patient.

### 2.2. Surgical Technique

RC and the expanded pelvic lymph node dissection procedure were performed in all patients included in the study. Longitudinal median incision was made from the level of the umbilicus to the symphysis pubis, which was approximately 10–12 cm. The steps of RC surgery were performed as previously described in the literature. For conduits, we preferred a 15–20 cm ileum segment. The ileum resection and reanastomosis of the residual bowel were performed by a single experienced general surgeon. After having the ileum segment, the left ureter was passed through the rectosigmoid tunnel to the opposite side. Then, both ureters were anastomosed to the ileum segment by the Bricker method [13]. A drainage catheter was placed in the operation site. The peritoneum, muscles, fascia, subcutaneous tissue, and skin were closed, and the procedure was concluded.

### 2.3. ERAS

ERAS is the term used to describe the concept of multimodal, perioperative interventions to improve postoperative outcomes [14]. The preoperative period includes the optimization of medical diseases, cessation of alcohol and cigarette consumption, improvement of nutritional status, avoidance of mechanical bowel preparation, avoidance of long fasting periods and carbohydrate loading, avoidance of long-acting agents in preanesthetic medication, and thromboembolism prophylaxis [15]. Prophylactic antibiotic applications should be initiated 1 hour before the incision and should be stopped 24 hours postoperatively. If there are risk factors for the development of infection or if the operation time is longer than 3 hours, antibiotic application can be extended up to 72 hours postoperatively [16]. Evidence from colorectal and RC studies suggests that ERAS anesthetic protocols should encompass the use of the thoracic epidural (T9–11), advise minimal opioid use (using fentanyl-based, short-acting opioids if needed), and add strategies for the prevention of hypothermia, hypoxemia, and hypovolemia [17]. If possible, the most minimally invasive approach should be selected. In addition, the drainage catheter should be removed as soon as possible [18]. In the postoperative period, it is recommended to start enteral feeding within the first 24 hours and encourage mobilization in the first 6 hours. With the aim of providing effective pain relief and minimizing adverse effects, especially those that are associated with opioids, multimodal, opioid-sparing analgesia combined with regional or local anesthesia is a key component of ERAS. Postoperative removal of nasogastric tubes is preferred because studies have shown that prolonged use may delay the onset of bowel movements [19–21].

### 2.4. Statistical Analysis

Data were analyzed using SPSS for Windows, version 25.0 (IBM SPSS, Armonk, NY, USA). The normal distribution of continuous variables was analyzed using the Shapiro–Wilk test and histograms. Continuous variables in the independent group were compared with the Mann–Whitney *U* test. Discontinuous variables in the independent group were compared with *t*-tests. Categorical variables were compared using chi-squared tests. A *p* value of <0.05 was considered significant.

## 3. Results

A total of 182 patients with primary muscle-invasive bladder urothelial carcinoma underwent RC with ileal conduit from November 2017 to December 2020. The ERAS and traditional groups contained 72 and 112 patients, respectively. No significant difference existed between the two groups in terms of age, gender, and body mass index. Moreover, no statistically significant differences were observed between the two groups with regard to preoperative plasma albumin levels (39.3 vs. 39.4 g/L, *p*=0.143) and clinical disease stage (*p*=0.570).  Table 1 provides the patient demographics and disease profile.

SEF in the traditional group was longer than that in the ERAS group. While enteral feeding was started for patients in the ERAS group on the first postoperative day as a standard, SEF was observed to start, on average, at 3.3 (2–7) days in the traditional group (*p* < 0.001). LOS (14.8 vs. 10.4 days, *p*=0.003), duration of postoperative antibiotic use (4.6 vs. 7.8 days, *p*=0.004), and time of first stool (4.4 vs. 2.9 days, *p*=0.011) were found to be significantly shorter in the ERAS group. It was also observed that ileus developed in 28 (25%) patients in the traditional group and 12 (16%) patients in the ERAS group. Although the percentage of ileus development was lower in the ERAS group, no statistically significant difference existed between the two groups (*p*=0.387). Both groups showed a decrease in plasma albumin levels in the second postoperative week. The postoperative and presurgical albumin levels were not statistically different (*p*=0.681). These data are given in Table 2.

## 4. Discussion

The primary purpose of our study was to evaluate the differences between the ERAS protocol group and the traditional group in terms of SEF, first stool time, LOS, rate of ileus, duration of antibiotic use, and postoperative plasma albumin levels in patients who underwent RC with ileal conduit. Ileal conduit is the most preferred type of urinary diversion in RC [5]. Therefore, we only examined patients with ileal conduits in this study. Although the ERAS protocol was first applied in gastrointestinal surgeries, it can now be successfully applied in many different surgical procedures [16, 17]. The ERAS protocol is now being used in RC, which is one of the major urological surgeries. The ERAS protocol for RC reduces LOS, morbidity, and healthcare costs [7, 10].

In a retrospective study performed by Hanna et al. [18] on 296 patients, it was reported that LOS significantly decreased in the ERAS protocol group (*p*=0.004). However, there was no significant difference in the patient group with high comorbidity. On the other hand, many studies have shown that LOS significantly decreased in patients who had the ERAS protocol RC [19, 20]. Similarly, in our study, there was a statistically significant decrease in terms of LOS in the ERAS group (*p*=0.003). The mean LOS was 10.44 days in the ERAS group and 14.79 days in the traditional group.

Since bowel resection and anastomosis are performed in RC with ileal conduit, prolongation at the time to start of bowel movements and ileus are common complications. One of the most feared complications is bowel anastomosis leak [21]. In a randomized controlled study by Vlad et al. [22], it was observed that the first stool time was shorter in patients who underwent the ERAS protocol after RC (two vs. five days, *p* < 0.001). The superiority of the ERAS protocol group in terms of postoperative ileus has not been demonstrated. Stimulation of the gastrointestinal system in the early postoperative period may shorten the first bowel movement time [23]. Another point is that the use of opioids for postoperative analgesia is restricted in the ERAS protocol. A known side effect of opioids is that they reduce intestinal mobility. Uncontrolled usage of opioids after surgery is also associated with prolonged bowel recovery time [24]. Similarly, in our study, we found that the first stool time was shorter in the ERAS group (*p*=0.011). Although there are various definitions of ileus in the literature, conditions such as oral diet intolerance, abdominal distention, and tenderness in physical examination, the need for the nasogastric tube was defined as ileus in this study. It was observed that ileus developed in seven (25%) patients in the traditional group and three (16%) patients in the ERAS protocol group. Although there was a lower rate of ileus in the ERAS protocol group, no statistically significant difference was found (*p*=0.387). In our opinion, the early onset of feeding may explain this difference.

The ERAS protocol standardized the use of prophylactic antibiotics during surgery. Prophylactic antibiotic applications in traditional perioperative patient care may differ between clinics. There are studies in the literature showing that the ERAS protocol does not differ significantly with other groups in terms of fever and infection development [25]. To date, there are not enough studies examining the effect of the ERAS protocol on the duration of antibiotic usage. We found that duration of antibiotic usage was shorter in the ERAS protocol group (*p*=0.004). This may have been caused by leaving the prophylaxis time to the surgeon's preference. While this issue was not investigated in our study, we hypothesize that the usage of antibiotics for a shorter time will reduce medical costs and allow us to avoid drug-related side effects.

Perioperative prolonged fasting may impair the nutritional status of patients. While intake of solid seedlings was restricted 6 hours before the operation in patients in the ERAS group, clear fluid intake was allowed up to 2 hours beforehand. We found that there was no certain standard for perioperative fasting in the traditional group. Plasma albumin levels are closely related to nutritional status in surgical patients. While albumin synthesis rate decreases in the operative phase, it increases in the postoperative phase if sufficient amino acid intake is provided [26]. Despite enteral feeding being initiated earlier in the ERAS protocol group, there was no significant difference between postoperative second week plasma albumin levels (mean 29.46 vs. 28.85 g/L, *p*=0.681).

Analyzing these data is limited by several weaknesses in our study. First of all, it is a retrospective case series with a limited number of patients (46). However, we believe that utilizing subjective data is more clinically relevant for practitioners and their patients. The number of patients in this study could have been increased, but the pandemic led to a decrease in the admission of patients to our outpatient clinic according to each symptom type, including gross hematuria, which is the most common symptom type in patients having bladder carcinoma. Finally, all of the ileum resection and bowel anastomosis were performed by a single experienced surgeon. While this provides standardization in the surgical technique, it makes the results less suitable for the wider community and limits external validity.

According to the results in our study, the ERAS protocol shortens the length of hospital stay, duration of antibiotic use, and time to first stool in patients who underwent RC with ileal conduit. There was no significant difference in the traditional group in terms of ileus, which is a common complication in gastrointestinal surgeries.

## Figures and Tables

**Table 1:** Patients demographics and disease profile.

			Traditional group	ERAS group	*P* value
Age, years	Mean		63.32 (±8.02)	66.94 (±8.01)	0.143
Gender	Male	Count	25 (89%)	16 (89%)	0.659
	Female	Count	3 (11%)	2 (11%)	
^*∗*^BMI, kg/m^2^	Mean		27.24 (±3.46)	25.75 (±3.17)	0.311
Clinical stage	≤T2	Count	24	15	0.570
	≥T3	Count	4	3	
^*∗∗*^Prealbumin, g/L	Mean		39.34 (±6.00)	39.39 (±5.86)	0.977

^*∗*^BMI, body mass index. ^*∗∗*^Prealbumin, preoperative serum albumin level.

**Table 2:** Postoperative data.

		Group				*P*
		Traditional group		ERAS group		
		Mean	Count	Mean	Count	
^*∗*^SEF, day		3.32 ± 1.49		1.00 ±0.00		<0.001
^*∗∗*^Defecation, day		4.43 ± 2.39		2.89 ± 1.81		0.011
^*∗∗∗*^LOS, day		14.79 ± 6.44		10.44 ± 4.64		0.003
^*∗∗∗∗*^Antibiotic, day		8.79 ± 5.17		4.61 ± 4.90		0.004
İleus	No		21 (75%)		15 (84%)	0.387
	Yes		7 (25%)		3 (16%)	
^*∗∗∗∗∗*^Postalbumin, g/L		28.85 ± 4.85		29.46 ± 5.35		0.681

^*∗*^SEF, time to start enteral feeding; ^*∗∗*^defecation, first stool time; ^*∗∗∗*^LOS, length of hospital stay; ^*∗∗∗∗*^antibiotic, duration of postoperative intravenous antibiotic use; ^*∗∗∗∗∗*^postalbumin, postoperative serum albumin level.

## Data Availability

The data used to support the findings of this study are available from the corresponding author upon request.
